# Bullous Prurigo Pigmentosa

**DOI:** 10.1515/biol-2019-0024

**Published:** 2019-07-10

**Authors:** Xinjun Wang, Chenchen Xu

**Affiliations:** 1 Department of dermatology, Guang’anmen Hospital, China Academy of Chinese Medical Science 100053 Beijing PR China; 2 Department of Dermatology, the People’s Hospital of Fenghua Ningbo, Zhejiang Province 315500 PR China

**Keywords:** Bullous prurigo pigmentosa, inflammatory dermatosis, pruritic erythematous plaques, diagnosis

## Abstract

Prurigo pigmentosa (PP) is an inflammatory dermatosis with unknown etiology. The clinical presentations of PP varies according to the stages of the disease. Rarely, the formation of numerous vesicles and bullae upon erythematous infiltrative plaques can be found during the entire clinical course. In the present case, a 29-year-old Chinese woman presented with a 6-year history of relapsing pruritic erythematous plaques and bulla on her neck, chest and back. Physical examination revealed multiple erythematous plaques and vesicles in combination with mottled pigmentation in a symmetrical distribution and reticular pattern on the nape of her neck, chest and back. Histological examination of the biopsy specimen collected from the bullous area of her chest indicated a lichenoid reaction with intraepidermal bulla. This inflammatory region is characterized by recruitment of lymphocytes, spongiosis, and a perivascular lymphohistiocytic infiltrate in the upper dermis. Direct immunofluorescence analysis for IgG, IgA, IgM and C3 was negative. The diagnosis of bullous prurigo pigmentosa was verified based on the clinical manifestation and pathological findings. Minocycline hydrochloride therapy (100mg/d) was initiated, and 3 weeks later the rash had completely disappeared, which resulted in pigmentation of the entire area. No recurrence was observed during the 4 years follow-up.

## 1 Introduction

Prurigo pigmentosa (PP) is an inflammatory dermatosis with unknown etiology. The clinical presentation of PP varies according to different stages of the disease. It is characterized by recurrent, pruritic, erythematous papules, which can develop into reticular hyperpigmentation on the back, chest and neck [1]. The lesions can develop a crusted and/or scaly form and can resolve spontaneously within a few weeks. Consequently, it is possible to observe the lesions from different stages in the same region. Rarely, the formation of numerous vesicles and bullae upon erythematous infiltrative plaques can be found throughout the clinical course [2].

## 2 Case presentation

**Figure 1:**
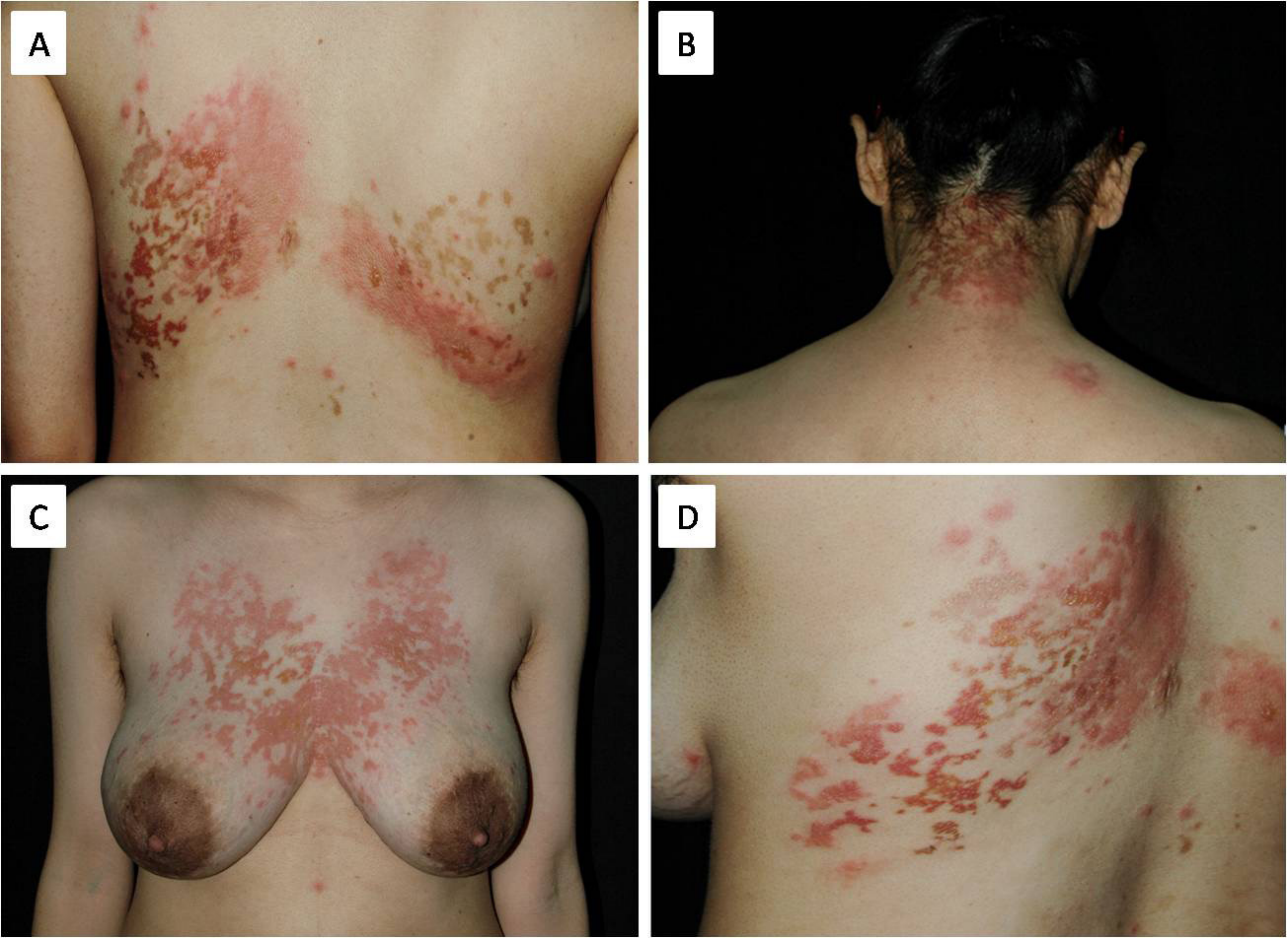
Multiple erythematous plaques and vesicles in combination with mottled pigmentation in a symmetrical distribution and reticular pattern on back (A, D), the nape of the patient’s neck (B) and chest (C).

**Figure 2:**
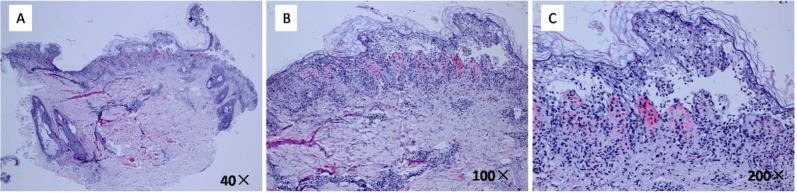
Histological examination of the biopsy specimen that was collected from the bullous area of the patient’s chest indicated a lichenoid reaction with intraepidermal bulla that was characterized by lymphocyte recruitment, spongiosis, and a perivascular lymphohistiocytic infiltrate in the upper dermis (A:40×; B:100×; C:200×).

A 29-year-old Chinese woman presented with a 6-year history of relapsing pruritic erythematous plaques and bulla on her neck, chest and back was admitted to our hospital. She had undergone treatment with topical steroids and oral antihistamines without appreciable benefit. Her medical record and family history were unremarkable. Following physical examination, the identification of multiple erythematous plaques and vesicles in combination with mottled pigmentation was evident. These structures were noted in a symmetrical distribution and reticular pattern and were localized on the nape of the patient’s neck, chest and back (**Figure. 1**). The results of the laboratory investigations, including liver, kidney, and thyroid function tests, blood glucose levels, and antinuclear body titers, were all within normal limits. Direct immunofluorescence analysis of IgG, IgA, IgM and C3 were negative. Histological examination of the biopsy specimen collected from the bullous area of the patient’s chest indicated a lichenoid reaction with intraepidermal bulla that was characterized by lymphocyte recruitment, spongiosis, and a perivascular lymphohistiocytic infiltrate in the upper dermis (**Figure 2**). The diagnosis of bullous prurigo pigmentosa was confirmed based on the clinicopathological findings. Therefore, minocycline hydrochloride therapy (100mg/d) was initiated and the erythematous plaques and bullous area on the neck, chest and back revealed a rapid pigmentation within the first week, whereas 3 weeks later the rash had completely disappeared leaving the entire area pigmented. No recurrence was observed after 4 years of follow-up.

**Informed consent**: Informed consent has been obtained from the patient included in this study

**Ethical approval**: The research related to human use has been complied with all the relevant national regulations, institutional policies and in accordance the tenets of the Helsinki Declaration, and has been approved by the ethical committee of People’s Hospital of Fenghua Ningbo.

## 3 Discussion

Women have been shown to be two or three times more susceptible to PP than men, where a majority of the cases reported were among Japanese females. The mean age of the disease onset is between 23 and 27 years [3]. Systemic conditions, including ketosis, diabetes, pregnancy, anorexia nervosa, atopic diathesis, rapid weight loss, *Helicobacter pylori* infection and exogenous factors, such as sweating, friction from clothing, and exposure to allergens, are considered as pathogenic and/or triggering factors of PP [4]. Certain case reports of bullous prurigo pigmentosa have been associated with diabetes mellitus and/or ketosis. However, the relationship between these factors and the pathogenesis of PP is still unclear. The case reported in the current study presented sudden onset of vesicles and erythematous plaques over the nape of neck, chest and back in the absence of endogenous or exogenous factors.

Histopathological findings of PP are unspecific and vary through different stages. Bullous prurigo pigmentosa is characterized by spongiosis, intraepidermal and subepidermal blisters with lymphocytic infiltration, and lichenoid eruption with release of eosinophils [4, 5]. It has been suggested that the spongiosis and liquefaction of the basal cell layer are possible causes for the formation of vesicles [2].

The differential diagnoses of bullous prurigo pigmentosa include confluent and reticulate papillomatosis, bullous lichen planus, dermatitis herpetiformis and bullous systemic lupus erythematosus. Confluent and reticulate papillomatosis is more likely to occur in overweight patients and presents with a reticular pattern at the periphery, which is formed by hyperpigmented scaly papules in the absence of pruritus. Bullous systemic lupus erythematosus is characterized by widespread, transient, non-scarring vesiculobullous eruption under the background of systemic lupus erythematosus [6]. Direct immune-fluorescence is considered the best method to diagnose dermatitis herpetiformis, which reveals granular IgA at the dermal-epidermal junction [7]. Bullous lichen planus always presents as tense and multilocular blisters with typical lesions of lichen planus. The histopathological findings were indicative of lichen planus-specific changes, whereas the direct and indirect immunofluorescence tests were negative [8].

Once diagnosed, successful treatment of PP can achieve a satisfying response and reduce significantly the incidence of relapse. The treatments include tetracyclines (doxycycline and minocycline), dapsone, sulfamethoxazole and macrolides. The utilization of minocycline (100-200mg/d) appears to be the most effective approach in the majority of the reported cases [9,10].
